# Pyramidal neurons in the superficial layers of rat retrosplenial cortex exhibit a late-spiking firing property

**DOI:** 10.1007/s00429-012-0398-1

**Published:** 2012-03-01

**Authors:** Tohru Kurotani, Toshio Miyashita, Marie Wintzer, Tomokazu Konishi, Kazuhisa Sakai, Noritaka Ichinohe, Kathleen S. Rockland

**Affiliations:** 1 Lab for Cortical Organization and Systematics, RIKEN Brain Science Institute, 2-1 Hirosawa, Wako, 351-0198 Japan; 2 ERATO, Okanoya Emotional Information Project, Japan Science and Technology Agency, 2-1 Hirosawa, Wako, 351-0198 Japan; 3 Faculty of Bioresource Sciences, Akita Prefectural University, Akita, 010-0195 Japan; 4 Department of Ultrastructural Research, National Institute of Neuroscience, Kodaira, 187-8551 Japan; 5 Picower Institute for Learning and Memory, Massachusetts Institute of Technology, Cambridge, MA 02139 USA

**Keywords:** Retrosplenial cortex, Pyramidal neuron, Firing property, Potassium channel, Late spiking

## Abstract

The rodent granular retrosplenial cortex (GRS) is reciprocally connected with the hippocampus. It is part of several networks implicated in spatial learning and memory, and is known to contain head-direction cells. There are, however, few specifics concerning the mechanisms and microcircuitry underlying its involvement in spatial and mnemonic functions. In this report, we set out to characterize intrinsic properties of a distinctive population of small pyramidal neurons in layer 2 of rat GRS. These neurons, as well as those in adjoining layer 3, were found to exhibit a late-spiking (LS) firing property. We established by multiple criteria that the LS property is a consequence of delayed rectifier and A-type potassium channels. These were identified as Kv1.1, Kv1.4 and Kv4.3 by Genechip analysis, in situ hybridization, single-cell reverse transcriptase-polymerase chain reaction, and pharmacological blockade. The LS property might facilitate comparison or integration of synaptic inputs during an interval delay, consistent with the proposed role of the GRS in memory-related processes.

## Introduction

The granular retrosplenial (GRS) cortex of rodents is an important structure in several networks involved in spatial learning and memory (Cooper and Mizumori 2001; Pothuizen et al. 2008; Garden et al. 2009; Vann et al. 2009; Aggleton et al. 2010). Consistent with this, lesion-behavior experiments have demonstrated that damage to the GRS results in specific impairments in spatial working memory (Keene and Bucci 2009), and selective activations in the GRS have been reported in spatial tasks using immediate early genes (Pothuizen et al. 2009). This aspect of GRS function has been attributed to its dense reciprocal connections with the hippocampal formation, with the idea that the two areas operate conjointly to support spatial memory (Mizumori et al. 2000). One recent report, corroborating earlier studies, found that hippocampal lesions produce marked reductions in the levels of immediate early gene proteins in the GRS, subsequent to behavioral tasks (Albasser et al. 2007). Several other recent investigations provide evidence that disrupting the dense reciprocal connections between the GRS and anterior thalamic nucleus results in a striking loss of synaptic plasticity in the superficial layers of the GRS (Garden et al. 2009; Wright et al. 2010).

The underlying substrates of GRS function are complex, and are likely to incorporate both intrinsic cellular specializations as well as network properties. Theta-rhythmic activity has been postulated to coordinate activity in distributed systems, including the GRS, during mnemonic processes (Kirk and MacKay 2003). Detailed information for the GRS, however, is largely limited to anatomical characterization of individual neuronal types (Vogt and Peters 1981; Wyss et al. 1990), and identification of the major inputs and outputs (Sripanidkulchai and Wyss 1986; van Groen and Wyss 1990; Wyss and van Groen 1992; Shibata et al. 2004; Aggleton et al. 2010).

A distinctive feature of the rodent GRS is an accentuated layer 2, consisting mainly of closely packed, callosally projecting small pyramidal neurons (Wyss et al. 1990; Ichinohe et al. 2008). In the rat, the apical dendrites of these neurons form prominent bundles, which co-localize with parvalbumin-positive dendrites (Ichinohe and Rockland 2002) and with patches of thalamic terminations (Shibata 1993).

As a step toward elucidating synaptic properties of this neuronal population, we recorded from slice preparations of the rat GRS to characterize intrinsic membrane characteristics. We found that the majority of pyramidal neurons in layer 2, and some in underlying layer 3, have a distinctive late-spiking (LS) firing pattern, where an initial rapid rise in membrane potential is followed by a slowly ramping depolarization that leads to an action potential firing near the end of a just-threshold current step. This LS property is unusual for pyramidal neurons, but has been previously reported for pyramidal neurons of rat perirhinal cortex (Beggs et al. 2000; McGann et al. 2001; Moyer et al. 2002).

We further established that the LS property is a consequence of delayed rectifier and A-type potassium channels. These were identified as Kv1.1, Kv1.4 and Kv4.3 by several corroborating techniques; namely, Genechip analysis, in situ hybridization, single-cell reverse transcriptase-polymerase chain reaction (RT-PCR), and pharmacological blockade.

## Materials and methods

### Slice preparation and recording of intrinsic membrane properties

Postnatal day 20–35 Wistar rats were deeply anesthetized with isoflurane and decapitated. The brain was quickly removed and immersed into chilled and oxygenated (95% O_2_ and 5% CO_2_) artificial cerebrospinal fluid (ACSF) containing (in mM): NaCl 126, KCl 3, NaH_2_PO_4_ 1.2, MgSO_4_ 1.3, CaCl_2_ 2.4, NaHCO_3_ 26, and glucose 10. Coronal or horizontal slices (300 μm thick) were prepared from GRS cortex using a Pro-7 vibrating microtome (Dosaka, Kyoto, Japan). After cutting, the slices were transferred to an interface-type chamber and perfused with oxygenated ACSF at 32–34°C for at least 1 h for recovery. Then one slice was selected and placed in a recording chamber continuously perfused with oxygenated ACSF (2 ml/min) at 27–30°C. Layer 2 of GRS cortex was easily visualized by virtue of its cell density, and neurons were targeted for recording on the basis of a pyramidal-like shape as visualized by infra-red differential interference contrast video microscopy (BX-50, Olympus, Tokyo, Japan). Whole-cell recordings were conducted with borosilicate patch pipettes (BF150-110-10, Sutter Instrument, Novato, CA, USA) filled with an internal solution containing (in mM): K-gluconate 150, NaCl 10, MgSO_4_ 5, HEPES 10 and EGTA 0.3, with 3 mg/ml biocytin and pH 7.3 adjusted with KOH. Current-clamp recordings were made by an Axoclamp 2B amplifier (Molecular Devices Corp., Sunnyvale, CA, USA) and intrinsic firing properties were investigated by injecting step-depolarizing currents (duration 1 s, amplitude ±10–200 pA). In some of the experiments, spontaneous postsynaptic responses were suppressed by perfusing 40 μM DNQX, 25 μM DL-APV and 20 μM bicuculline methiodide. To confirm that the firing pattern of the neurons was unchanged at 36°C, intracellular recordings from GRS layer 2 neurons were performed using sharp glass microelectrodes (containing 2 M K-gluconate and 10 mM KCl, resistance >150 MΩ) in a Haas type interface chamber. In all electrophysiological recordings, the electrode resistance was effectively canceled out using a bridge balance circuit equipped in Axoclamp 2B.

After the recording, the cells were filled with biocytin by diffusive loading through the patch pipette for 10–15 min. The patch pipette was slowly retracted from the cell after the filling so that the cell membrane was successfully resealed. One to eight neurons were filled per slice, and the slice was then transferred back to the interface-type chamber for more than 1 h for completion of biocytin transport. Slices were fixed overnight at 4°C in 4% paraformaldehyde (PFA) containing 0.3% picric acid, washed four times for 10 min each in phosphate buffered saline (PBS) at room temperature, and treated with 1% H_2_O_2_ in 0.1 M PBS for 20 min. After four rinses of 5 min each in 0.1 M PBS, the slices were incubated overnight in an avidin–biotin complex (one drop of each reagent for 7 ml of 0.1 M PBS containing 1% TritonX; ABC Elite kits, Vector Laboratories, Burlingame, CA, USA) at room temperature. Next day, sections were rinsed four times in 0.1 M phosphate buffer (PB) for 10 min each, and DAB histochemistry (25 mg/50 ml, in 0.1 M PB) was performed with the addition of 0.03% nickel ammonium sulfate. In some experiments, the same slice preparation, recording and staining procedures were carried out for the pyramidal neurons in layers 3 and 5 of GRS, layer 2 of the barrel field cortex (BF) of adult rats for comparison.

To compare the dendritic branching pattern, the stained pyramidal neurons in layer 2 of GRS and of BF were investigated by Sholl analysis. Labeled pyramidal neurons were analyzed using Neurolucida (Micro-BrightField Inc, Colchester, VT). A series of concentric circles, with radius increasing by 10 μm increments, were drawn starting from the center of the cell soma. Then the number of intersections made by the particular dendrite with each circle was counted using NeuroExplorer (Micro-BrightField Inc), and plotted against the distance from the soma. For statistical analyses of data obtained from the electrophysiological and morphological experiments, Student’s *t* test was employed unless otherwise mentioned.

### Microarray data

From a parallel investigation involving rat GRS (Miyashita et al. 2010), we had microarray data for genes which are highly and specifically expressed in GRS layer 2. Briefly, concerning the criteria for gene selection, we compared gene expression profiles for layer 2 of GRS, layer 5 of GRS, and layer 2 of the somatosensory barrel cortex at postnatal day 28. Significance in expressional change between layers 2 of GRS and BF was tested gene-wise using paired *t* test on perfect match (PM) cell data of microarray (GeneChip, Rat Expression 230 2.0 Array; Affymetrix, Santa Clara, CA). Among the corresponding PM data of a gene, all the data that were out of the plausible signal range (Konishi 2004, 2008), and those within the detected area but caused by dust contamination (Konishi 2006), were removed. Then, *t* test was performed by cell-wise comparison, using a threshold of 0.01. Genes were further selected that showed three times higher expression levels in layer 2 than in layer 5 of GRS (Table 1). Full details are given in Miyashita et al. 2010.

**Table 1:** List of Kv channel genes that were highly expressed in GRS layer 2

HGNC name	IUPHAR name	Ratio (GRSL2/BFL2)	*P* value	Ratio (GRSL2/GRSL5)
Kcnd3	**Kv4.3**	3.55	5.05E − 17	**8.65**
Kcna4	**Kv1.4**	4.33	1.67E − 15	**4.24**
Kcns1	Kv9.1	2.27	1.58E − 07	2.53
Kcnd2	Kv4.2	1.82	5.46E − 08	2.25
Kcna1	**Kv1.1**	**5.05**	5.37E − 22	1.57
Kcnd2	Kv4.2	1.22	4.35E − 05	1.26
Kcnc1	Kv3.1	1.80	3.12E − 10	1.22
Kcng2	Kv6.2	1.25	1.49E − 03	1.02
Kcnb1	Kv2.1	1.24	6.98E − 06	0.93
Kcns3	Kv9.3	2.39	1.62E − 12	0.92
Kcnc3	Kv3.3	1.40	1.01E − 03	0.88
Kcnab1	Kvb1.3	3.54	9.62E − 19	0.77

The expression level of each gene in GRS layers 2, 5 and BF layer 2 was calculated as *z* score. Then significant difference in expression levels of each gene in GRS layer 2 and BF layer 2 was calculated by Welch’s paired two-sided *t* test, and the *P* values were determined (Konishi 2004, 2006, 2008). Kv channel genes, having expression ratio >1 and *P* < 0.01 in GRS layer 2 compared to BF layer 2, were selected. The genes are sorted by the expression ratio of those in GRS layer 2 to in GRS layer 5. Note that Kv4.3 and Kv1.4 genes are much more highly expressed in GRS layer 2 than in GRS layer 5 (ratio; 8.65 and 4.24, respectively). It should be also noted that Kv1.1 was highly expressed in GRS layer 2, compared to BF layer 2 (ratio; 5.05)

*HGNC* HUGO Gene Nomenclature Committee, *IUPHAR* International Union of Pharmacology

### In situ hybridization for Kv1.4

PCR primers for Kv1.4 (5′-CATAATTGTGGCGAACGTG-3′ and 5′-TTTTGAAAGATTCGGCTGCT-3′) were designed based on the rat cDNA sequence of Kv1.4 (GenBank No. NM_012971). The DNA fragments were produced by RT-PCR from rat brain cDNA. PCR fragments were ligated into the pGEMt-easy (Promega, Madison, WI) vector. The plasmids were extracted and linearized by *Asp718* or *Xho1* before being used for the template of antisense or sense probes. The digoxigenin (DIG)-dUTP labeling kit (Roche, Basel, Switzerland) was used for in vitro transcription.

Two adult rats were used for in situ hybridization for Kv1.4 mRNA. Animals were anesthetized with Nembutal intraperitoneally (100 mg/kg), and perfused transcardially, in sequence, with 0.9% NaCl and 0.5% NaNO_2_ for 1 min, and 4% PFA in 0.1 M PB for 10 min. Brains were removed and postfixed in the same fixative for 2 h, and then immersed into 30% sucrose in 0.1 M PB until sinking (20–40 h). Sections were cut (in the coronal plane, at 30 μm thickness) using a sliding microtome. Sections were washed in 0.1 M PB, and again postfixed with 4% PFA in 0.1 M PB for 10 min. After washing in 0.1 M PB, sections were treated with 1 μg/mL proteinase K for 10 min at 37°C, acetylated, then incubated in hybridization buffer containing 0.5–1.0 μg/mL DIG-labeled riboprobes at 60°C over night. The sections were sequentially treated for 15 min at 55°C in 2× standard sodium citrate (SSC)/50% formamide/0.1% *N*-lauroylsarcosine, twice; for 30 min at 37°C in RNase buffer (10 mM Tris–HCl, pH 8.0, 1 mM EDTA, 500 mM NaCl) containing 20 μg/mL RNase A (Sigma, St. Louis, MO, USA); for 15 min at 37°C in 2× SSC/0.1% *N*-lauroylsarcosine, twice; for 15 min at 37°C in 0.2× SSC/0.1% *N*-lauroylsarcosine, twice. The hybridized probe was detected by alkaline phosphatase-conjugated anti-DIG antibody with DIG detection kits (Roche Diagnostics, Basel, Switzerland). Controls with sense riboprobes showed no hybridization signal.

### Single-cell RT-PCR experiment

To assess potassium channel mRNAs across individual LS pyramidal neurons, single-cell RT-PCR experiments were performed. Recording pipettes were prepared as above, except that an RNase inhibitor (Takara, Otsu, Japan, final concentration, 0.5 U/μl) was added to the pipette solution. Pipettes were filled with 5 μl of the solution. Directly after patch-clamp recording, the contents of the cell including the nucleus were aspirated into the patch electrode. The small size of LS neurons made it difficult to obtain enough cytoplasm for single-cell RT-PCR without including the nucleus. However, by employing the intron-spanning assay, it was possible to effectively differentiate between genome-derived and mRNA-derived signals (Liss and Roeper, 2004). The electrode tip was broken off into a reaction tube containing 5 μl diethylpyrocarbonate treated water containing RNase inhibitor (0.5 U/μl, RNase OUT, Takara), and the tubes were briefly stored on ice until use.

#### Reverse transcription (RT)

mRNA was reverse transcribed with the Superscript III CellsDirect cDNA Synthesis System kit (Invitrogen, San Diego, CA, USA). The reverse transcription mixture containing the cell contents, 1 μl Oligo(dT)20 (50 mM) and 0.5 μl dNTP mix (10 mM) was first heated to 70°C for 5 min and incubated on ice for 2 min. Single-strand cDNA synthesis was carried out at 50°C for 50 min, after the addition of 3 μl 5× RT buffer, 0.5 μl RNase OUT (40 U/μl), 0.5 μl DTT (0.1 M) and 0.5 μl Superscript III RT (200 U/μl) in a final volume of 15 μl. This reaction was terminated at 85°C for 5 min. Before PCR amplification, 1 μl of RNase (2 U/μl) was added and the samples incubated at 37°C for 20 min. Ten microliters of RT products were used for following PCR amplification.

#### PCR amplification

Multiplex PCR conditions were optimized using total RNA purified from rat brain. Primer pairs were designed to locate on different exons separated by introns to prevent amplification of genomic DNA. Under these conditions, subsequent gel analysis did not detect nonspecific products. Controls for contaminating artifacts using sterile water instead of DNA, and PCR done on samples without reverse transcriptase did not detect any product. A multiplex two-round single-cell PCR was carried out for simultaneous detection of vesicular glutamate transporter 1 (VGluT1), GABA_A_ receptor α1 subunit, and voltage-activated potassium channels Kv1.1, Kv1.2, Kv1.4, Kv3.1 and Kv4.3. GABA_A_ receptor α1 subunit and β-actin were used as a positive control.

The first amplification round consisted of 15 min hot start at 95°C, followed by 40 cycles (94°C for 30 s, 57°C for 1.5 min and 72°C for 1 min). All genes were simultaneously amplified in a single tube containing 10 μl of the RT product, 250 nM of each of the outer primers, 40 μM of each dNTPs, 2.5 U Ex Taq Hot Start DNA Polymerase (Takara) and 1× PCR buffer (Takara) in a final volume of 50 μl.

A second round of PCR consisted of 15 min hot start at 95°C, followed by 40 cycles (94°C for 30 s, 55°C for 1 min and 72°C for 1 min), and terminated at 72°C for 7 min. In this round, each gene was individually amplified in a separate test tube containing: 1 μl of the first PCR product (template), 250 nM of each nested (inner) primers, 30 μM of each dNTP, 2 U Ex Taq Hot Start DNA Polymerase (Takara) and 1× PCR buffer (Takara) in a final volume of 30 μl. The products of the second PCR were analyzed by 2% agarose gel electrophoresis (see Fig. 9 for example). Primers used for the experiment are shown in Table 2.

**Table 2:** List of primers used for the single-cell RT-PCR experiment

Kv1.1 outer	Forward: 5′-TGCCCATGAAGTAGTCTGTG-3′
	Reverse: 5′-ATCCACTTCTGAAGGTCAGG-3′
Kv1.1 inner	Forward: 5′-CGTGGAACACCATGTAACAG-3′
	Reverse: 5′-AGGGGTCAGGATTGGTTT-3′
Kv1.2 outer	Forward: 5′-GGGGACAGAGTTAGCTGAGA-3′
	Reverse: 5′-TCCCTCCTGTATCTCCATGT-3′
Kv1.2 inner	Forward: 5′-GTCCAGACACTCCAAAGGTC-3′
	Reverse: 5′-TCTCCCGGTGGTAGAAGTAG-3′
Kv3.1 outer	Forward: 5′-CCTGCTGTGACTGTATGCTC-3′
	Reverse: 5′-CCTGAACTGGAGGGACTCT-3′
Kv3.1 inner	Forward: 5′-GGAGGTCAGGGACTAAGGAT-3′
	Reverse: 5′-CACTGGAGCTACACACCAAG-3′
Kv1.4 outer	Forward: 5′-GTCAGTTGCCCATACCTACC-3′
	Reverse: 5′-CTCGGGACCACCTTTACTAT-3′
Kv1.4 inner	Forward: 5′-AAGAAGGGGTCAAGGAGTCT-3′
	Reverse: 5′-TAATGCCTCCCTCTTCTCC-3′
Kv4.3 outer	Forward: 5′-AAGATGCCTTGAGGTCTGAG-3′
	Reverse: 5′-AGGATGAAGACAGGGAGACA-3′
Kv4.3 inner	Forward: 5′-AGTGAGCCTCAGGGTTAGTG-3′
	Reverse: 5′-CAAAACACCAGGACTCCTCT-3′
β-actin outer	Forward: 5′-ACACGGCATTGTAACCAACT-3′
	Reverse: 5′-CATTGCCGATAGTGATGACC-3′
β-actin inner	Forward: 5′-AGAAGATTTGGCACCACACT-3′
	Reverse: 5′-CCATCTCTTGCTCGAAGTCT-3′
GABA_A_ α1 outer	Forward: 5′-ACGACCGTTCTGACCATGACAACCT-3′
	Reverse: 5′-AAAGATTCCAAATAGCAGCGGAAAG-3′
GABA_A_ α1 inner	Forward: 5′-CTCCTACAGCAACCAGCTATACCC-3′
	Reverse: 5′-GCGGTTTTGTCTCAGGCTTGAC-3′
VGluT1 outer	Forward: 5′-GGCCCCTCCCTTAGAACG-3′
	Reverse: 5′-CCTCCGATGGGTACGATGATA-3′
VGluT1 inner	Forward: 5′-CCTTTTGCGGTTCCTATGC-3′
	Reverse: 5′-AATGTATTTGCGCTCCTCCTC-3′

### Pharmacology

As blockers of Kv channels, we used dendrotoxin-K (DTX-K) (100 nM for Kv1.1), CP-339818 (1–3 μM for Kv1.4) and nicotine (100 nM for Kv4.3). Drugs were bath administrated after recording intrinsic membrane and firing properties of layer 2 pyramids in normal ACSF, and changes in the firing pattern were monitored at 0.5–1 min intervals over 10–15 min by applying 70–110 pA depolarizing current pulses.

## Results

Layer 2 in the rat GRS is densely populated by small pyramidal neurons and was readily identified by differential interference contrast microscope. Whole-cell recordings were made under current-clamp condition in pyramidal neurons to record passive membrane and intrinsic firing properties in response to depolarizing and hyperpolarizing step current injections (duration = 1 s, except for test recordings of 5–8 s). To evaluate laminar specificity of firing properties, we compared recordings from pyramidal neurons in the rat GRS layer 3, GRS layer 5, layer 2 of BF cortex, and, with smaller sample sizes, from the rat presubiculum and perirhinal cortex. Neurons with resting membrane potential more negative than −55 mV and overshooting action potentials were selected for further analyses. Properties of passive membrane and action potential for the recorded neurons are indicated in Table 3.

**Table 3:** Properties of LS pyramidal neurons in GRS layers 2 and 3, and RS pyramidal neurons in GRS layer 5 and BF layer 2

	Soma size (minor and major axes, μm)	*V*_rest_ (mV)	*R*_in_ (MΩ)	AP threshold (mV)	AP height (mV)	AP half width (ms)
GRS L2 (*n* = 130)	(11 ± 0.31) × (18 ± 0.47)	−75 ± 0.62	420 ± 15	−35 ± 0.85	93 ± 1.0	1.9 ± 0.06
GRS L3 (*n* = 32)	(10 ± 0.50) × (13 ± 0.48)**	−76 ± 0.97	450 ± 24	−36 ± 0.84	93 ± 2.3	1.7 ± 0.07
GRS L5 (*n* = 14)	(17 ± 0.81)** × (25 ± 1.3)**	−68 ± 1.2**	90 ± 13**	−39 ± 1.9	96 ± 2.4	1.8 ± 0.12
BF L2 (*n* = 21)	(12 ± 0.33) × (19 ± 0.81)	−74 ± 0.98	130 ± 6.5**	−40 ± 1.9*	100 ± 2.5*	2.5 ± 0.15**

Numbers are presented as mean ± SEM. Using a camera lucida, the soma shape of pyramids was approximated by a triangle or a diamond, and then the length of minor and major axes was measured. Significant differences between GRS L2 neurons and the other neuron groups are indicated as asterisks (*P < 0.05 and **P < 0.01). Numbers without the asterisks were not significantly different from those for GRS L2 neurons

**Fig. 1:**
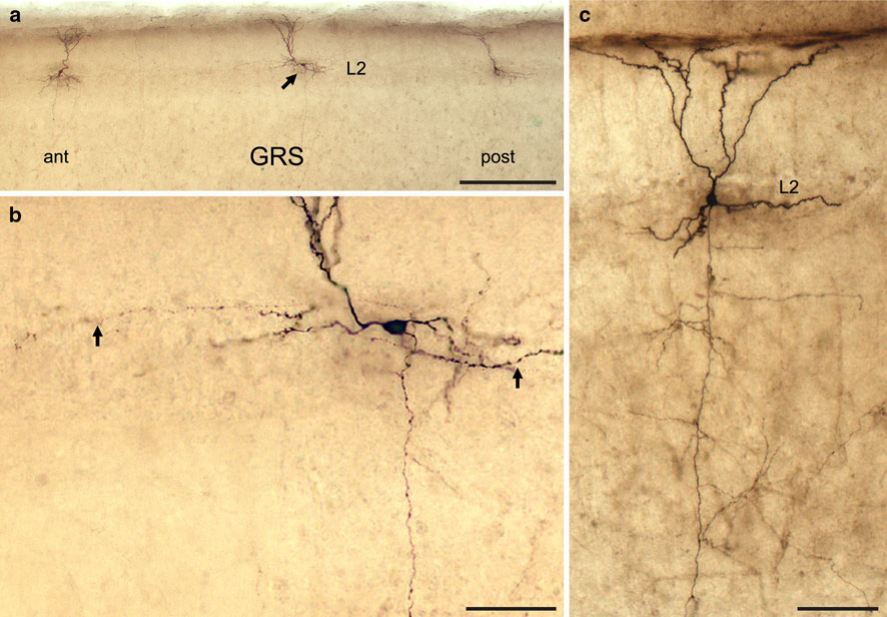
Biocytin-filled layer 2 pyramidal neurons in rat GRS cortex. **a** Low magnification view of three biocytin-filled neurons in layer 2. Horizontal slice (300 μm in thickness) from postnatal day 31 Wistar rat. *ant* anterior, *post* posterior, *scale bar* 300 μm. **b** Higher magnification view of the middle neuron (*arrow*) in **a**. *Two arrows* indicate horizontal axon collaterals largely within layer 2. *Scale bar* 50 μm. **c** Another example of a layer 2 neuron. *Scale bar* 100 μm

**Fig. 2:**
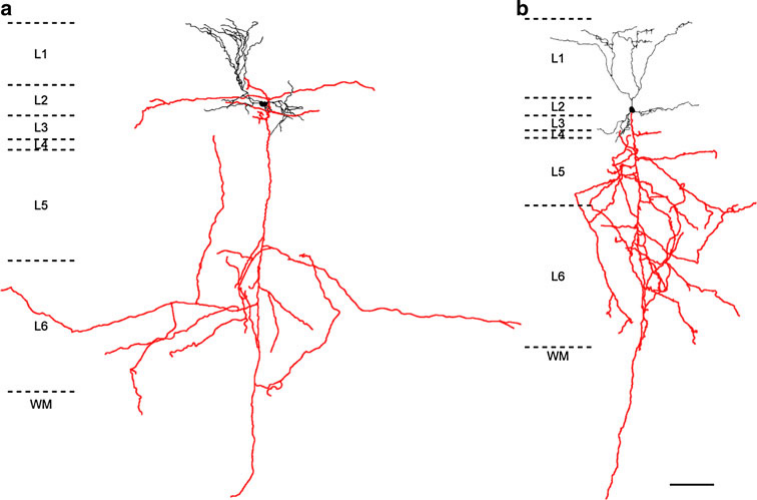
Neurolucida reconstruction of biocytin-filled neurons. **a** GRS L2 neuron indicated in Fig. 1b. The cell body and dendrites are shown in *black*, and the axon is shown in *red*. L1–L6 represent layers 1–6, respectively, and *dashed lines* indicate the borders between them. WM represents white matter. **b** Similar to **a**, but corresponding to GRS LS neuron in Fig 1 **c**. Axon reconstructions are necessarily limited to the portion contained within the 300 μm slice and are therefore not complete. (Same holds for the reconstructions shown in Figs. 4, 5, 6.) *Scale bar* 100 μm is common to **a** and **b**

Biocytin filling confirmed that the LS neurons were pyramidal. That is, dendrites were studded with spines and an apical dendrite was visible, extending into upper layer 1 (Figs. 1, 2). The apical dendrite was typically unbranched in its proximal portion, or had only 2–3 main bifurcations in layer 1c.

A more extensive tuft formed distally in layers 1a and 1b. Basal dendrites within layer 2 and/or 3 were prominently studded with spines. Slight morphological variations were evident, as described by previous Golgi studies (Vogt and Peters 1981; Fig. 5 in Wyss et al. 1990). Axon collaterals occurred in layers 1–6, being more abundant in layer 5 and 6 in our material. Long axonal segments could be followed up to about 350 μm from the soma, especially in layers 1 and 2. These had evidently been cut by the slicing procedure, and probably extended further. The average cell body size of layer 2 pyramids was significantly smaller than that of layer 5 pyramids in GRS (*P* < 0.01 for both of the minor and major axes, Table 3).

Subdivisions of layer 1 are determined following the criteria of Vogt et al. 1981. Layer 1a is subjacent to the pia and layer 1c is above layer 2. Layer 1b is identified by approximation as the middle sublayer.

### Firing and intrinsic membrane properties

#### GRS layer 2 pyramidal neurons

**Fig. 3:**
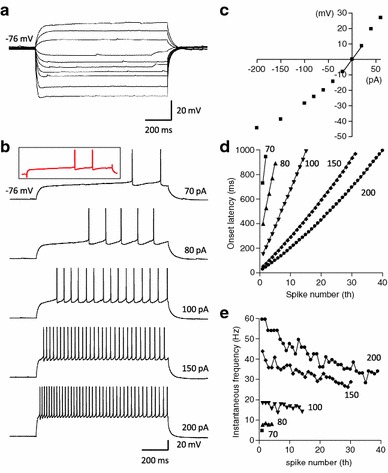
A representative example of passive membrane and firing properties of a layer 2 pyramidal neuron of GRS. **a** Voltage responses to hyperpolarizing and sub-threshold depolarizing current injections (intensity, −200–60 pA, duration, 1 s). **b** Voltage responses to supra-threshold depolarizing current injections. The current intensity is indicated at the end of each trace. This neuron had a resting membrane potential of −76 mV. Note that onset of the first action potential is substantially delayed from the initiation of the current injection. *Inset* red trace indicates a representative example of LS firing pattern recorded at 36°C from another GRS L2 neuron. **c** Current–voltage (I–V) relationship measured 800 ms after the onset of step currents in **a**. The input resistance of this neuron was 417 MΩ. **d** Onset latency of the first to *n*th spikes in response to the various intensities of the step current shown in **b**. Each* symbol* represents a different injection current intensity; *squares*, *triangles*, *inverted triangles*, *diamonds* and *circles* represent 70, 80, 100, 150 and 200 pA, respectively. **e** Inter-spike interval between *n*th to (*n* + 1)th spike, calculated from the onset latency data. *Symbols* are the same as those in **d**

Of 138 layer 2 pyramids recorded in GRS, 130 neurons (94%) showed a distinctive LS firing pattern. This was characterized by an initial rapid rise in membrane potential followed by a slowly ramping depolarization. Layer 2 LS neurons demonstrate a significantly hyperpolarized resting membrane potential (−75 ± 0.62 mV, *n* = 130, Fig. 3a; Table 3) compared with that of GRS layer 5 neurons (*P* < 0.01, Table 3). Voltage responses of LS neurons to hyperpolarizing current steps demonstrated little or no sag at the initial hyperpolarization phase, suggesting a weak expression of hyperpolarization-activated cation channels in these neurons (Fig. 3a). The first action potential occurred near the end of a just-suprathreshold current step (Fig. 3b). LS neurons have a very high input resistance (420 ± 15 MΩ, Fig. 3c; Table 3). This implies that LS neurons have a small cell body, as reported by others (Wyss et al. 1990) and confirmed in our morphological observation (Figs. 1, 2; Table 3). Because of a high input resistance, the threshold level was not always sharply determined. For 15 neurons in which we could sharply determine the threshold, the latency of the first spike was 809 ± 32.5 ms. As the intensity of the depolarizing current increased, the latency of the first spike shortened, but there was still a delay in the latency of the first spike from the time of current onset (Fig. 3b, d). Spike trains evoked by a stronger current pulse (>200 pA) exhibited a slight accommodation of firing. The instantaneous firing frequency was measured from the time difference between the *n* and (*n* + 1)th spikes (Fig. 3e). In response to a strong depolarizing current injection, the maximum instantaneous firing frequency was observed between the first and the second spikes, reaching a value of more than 50 Hz. From this, the frequency gradually diminished to 30–40 Hz at the end of the current step (Fig. 3e, filled circle). It was confirmed that the LS firing pattern could be recorded at 36°C (*n* = 5, Fig. 3b, inset).

#### GRS layer 3 pyramidal neurons

**Fig. 4:**
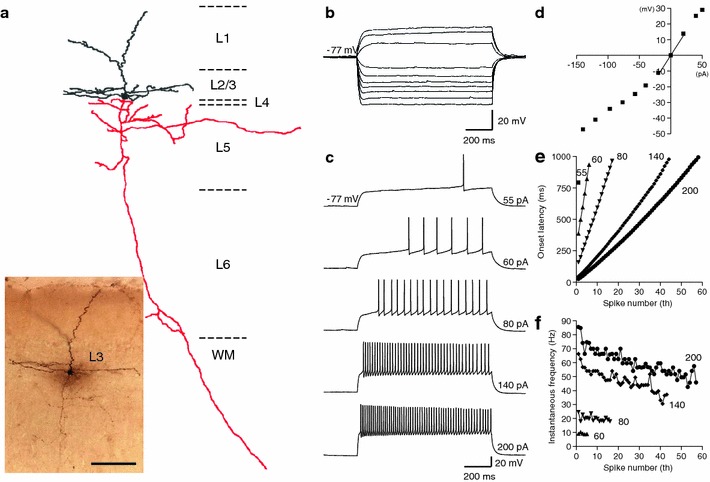
Layer 3 pyramidal neurons in GRS show similar firing and intrinsic membrane properties to layer 2 pyramids. **a** A biocytin-filled layer 3 pyramidal neuron and its Neurolucida reconstruction. The cell body and dendrites are shown in* black*, and the axon is shown in* red*.* Scale bar*, 100 μm. **b** Voltage responses to hyperpolarizing and sub-threshold depolarizing current injections (intensity, −140 to 50 pA, duration, 1 s). **c** Voltage responses to supra-threshold depolarizing current injections. The current intensity is indicated at the end of each trace. This neuron had a resting membrane potential of −77 mV. **d** I–V relationship measured 800 ms after the onset of step currents in (**b**). The input resistance of this neuron was 625 MΩ. **e** Onset latency of the first to* n*th spikes in response to the various intensities of the step current shown in (**c**). Each* symbol* represents a different injection current intensity;* square, triangle, inverted triangle, diamond* and* circle* represent 55, 60, 80, 140 and 200 pA, respectively. **f** Inter-spike interval between* n*th to (*n* + 1)th spike, calculated from the onset latency data.* Symbols* are the same as those in (**e**)

Pyramidal neurons in layer 3 of rat GRS were morphologically distinct from the LS neurons in layer 2. Their cell body was smaller than that of layer 2 LS neurons (Table 3; cf. Figs. 1, 2, 4a), and they had distinctly flat basal dendrites (extending straightly and parallel to the layer borders; Fig. 4a, see also Fig. 5 in Wyss et al. 1990). Despite the morphological difference, 32 of 34 neurons recorded in upper layer 3 had the same LS firing property as layer 2 neurons (Fig. 4b–f; Table 3). The remaining 2 showed a regular-spiking (RS) firing pattern.

**Fig. 5:**
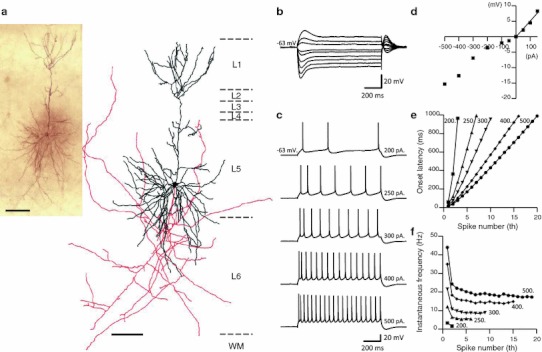
A representative layer 5 pyramidal neuron in GRS, demonstrating the RS firing property. **a** Biocytin-filled layer 5 pyramidal neuron and its Neurolucida reconstruction. The cell body and dendrites are shown in* black*, and the axon is shown in* red*.* Scale bars*, 100 μm. **b** Voltage responses to hyperpolarizing and sub-threshold depolarizing current injections (intensity, −500 to 150 pA, duration, 1 s). **c** Voltage responses to supra-threshold depolarizing current injections. The current intensity is indicated at the end of each trace. This neuron had a resting membrane potential of −63 mV. **d** I–V relationship measured 800 ms after the onset of step currents in (**b**). The input resistance of this neuron was 51 MΩ. **e** Onset latency of the first to* n*th spikes in response to the various intensities of the step current shown in (**c**). Each* symbol* represents a different injection current intensity: * square, triangle, inverted triangle, diamond* and * circle* represent 200, 250, 300, 400 and 500 pA, respectively. **f** Inter-spike interval between* n*th to (*n* + 1)th spike, calculated from the onset latency data.* Symbol*s are the same as those in (**e**)

#### Pyramidal neurons in layer 5 of GRS and layer 2 of BF

Consistent with many previous reports (Connors et al. 1982; McCormick et al. 1985; Sutor and Hablitz 1989; Mason and Larkman 1990; Steriade et al. 1993; Cho et al. 2004; Otsuka and Kawaguchi 2008), we found that most of the tested pyramidal neurons in layer 5 of GRS (74%, 14 of 19, Fig. 5a) demonstrated a RS firing pattern. Compared with GRS pyramidal neurons in layer 2, those in layer 5 of GRS had a more positive resting membrane potential (−68 ± 1.2 mV, Fig. 5b, c; Table 3), and a much lower input resistance (90 ± 13 MΩ, Table 3; Fig. 5d). The remaining 5 neurons in layer 5 of GRS showed an intrinsically bursting firing pattern. Even with a depolarizing current just above threshold, the latency of the first spike was 83.4 ± 17.2 ms, and significantly shorter than that of LS neurons (*P* < 0.01, Table 3). With increasing intensity of the depolarizing current, the latency of the first spike shortened, in marked contrast with LS neurons (Fig. 5e). These neurons showed adaptation during repetitive firing and lower maximum firing frequencies (Fig. 5f).

**Fig. 6:**
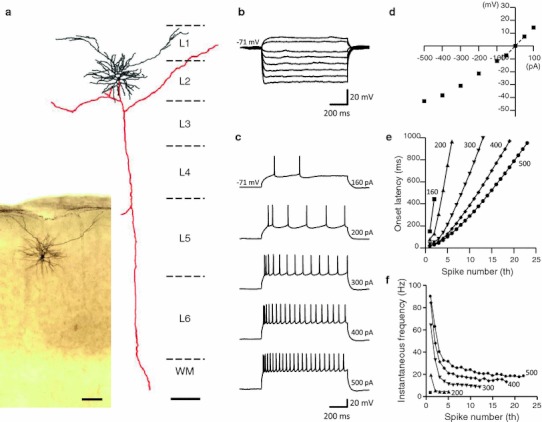
A representative layer 2 pyramidal neuron in BF, with a typical RS firing property. **a** Biocytin-filled layer 2 pyramidal neuron and its Neurolucida reconstruction. The cell body and dendrites are shown in *black*, and the axon is shown in *red*. *Scale bars* 100 μm. **b** Voltage responses to hyperpolarizing and sub-threshold depolarizing current injections (intensity, −500–100 pA, duration, 1 s). **c** Voltage responses to supra-threshold depolarizing current injections. The current intensity is indicated at the end of each trace. This neuron had a resting membrane potential of −71 mV. **d** I–V relationship measured 800 ms after the onset of step currents in (**b**). The input resistance of this example neuron was 146 MΩ. **e** Onset latency of the first to *n*th spikes in response to the various intensities of the step current shown in (**c**). Each* symbol* represents a different injection current intensity: *squares*, *triangles*, *inverted triangles*, *diamonds* and *circles* represent 160, 200, 300, 400 and 500 pA, respectively. **f** Inter-spike interval between* n*th to (*n* + 1)th spike, calculated from the onset latency data. Symbols are the same as those in (**e**)

As to the pyramidal neurons in BF layer 2, all of the cells tested (*n* = 21) demonstrated a RS firing pattern and the average input resistance showed an intermediate value (130 ± 6.5 MΩ, Fig. 6b–d; Table 3) between layer 2 and layer 5 pyramids in GRS. Interestingly, the average resting membrane potential of layer 2 pyramids in BF was −74 ± 0.98 mV, close to that of GRS pyramids in layers 2 and 3. Thus, despite differences in the firing properties, there may be common factors underlying the resting membrane potential for these populations. They had a shorter average latency for the initial AP firing (120 ± 15 ms, for a just-suprathreshold current, Fig. 6c) than that of LS neurons (*P* < 0.01). With increasing intensity of the depolarizing current, the latency of the first spike shortened, in marked contrast with LS neurons (Fig. 6e). These neurons showed adaptation during repetitive firing and lower maximum firing frequencies (Fig. 6f).

Biocytin fills confirmed that neurons recorded in layer 5 of the GRS and layer 2 of BF showed standard pyramidal cell morphologies (Figs. 5a, 6a, respectively).

#### Sholl analysis of layer 2 neurons in GRS and BF

**Fig. 7:**
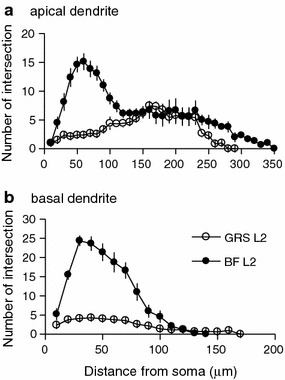
Sholl analysis of the apical and basal dendrites of layer 2 pyramidal neurons in GRS and BF. **a** The number of intersections of concentric circles made by the apical dendrites of GRS layer 2 (*open circles*) and of BF layer 2 neurons (*filled circles*), plotted against the distance from soma. **b** Similar to **a**, but for the basal dendrites

Distinctive dendritic morphology of layer 2 pyramidal neurons in GRS, as compared with that of pyramids in layer 2 of BF, was revealed by Sholl analysis (Fig. 7). Eight biocytin-filled RS pyramids in BF and 23 filled LS pyramids in GRS were selected for Sholl analysis. For BF layer 2 neurons, the number of intersections for apical dendrites had the highest values (about 15) at 50–60 μm from the soma (proximal dendritic zone), and gradually decreased until 350 μm more distally. In contrast, for GRS layer 2 neurons, the number of intersections of the apical dendrites was smaller (Fig. 7a), reaching highest values (about 6) at 160–170 μm from the soma (distal dendritic zone, corresponding to layer 1A). Dendritic branching was also less for the basal dendrites of GRS LS neurons than for those of BF RS neurons (Fig. 7b).

### The LS property is attributed to potassium channels

#### Microarray data

We identified two genes coding for different types of potassium channels, Kv1.4 and Kv4.3, that were highly expressed in GRS layer 2. (Layer 3 neurons were not investigated.) The expression ratio of these two genes in comparison with that in GRS layer 5 was 4.24 and 8.65, respectively, and was much higher than that of the other potassium channels (less than 2.53, Table 1). From these microarray data, we selected Kv1.4 and Kv4.3 channels for further analyses.

#### In situ hybridization

**Fig. 8:**
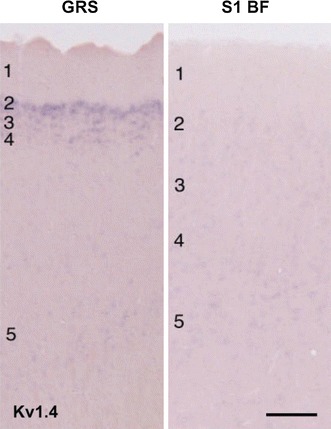
In situ hybridization for Kv1.4 mRNA in GRS and BF cortices. Cortical layers numbered at *left*. *Scale bar* 300 μm

By in situ hybridization, we confirmed that the Kv1.4 signal was highly expressed in the superficial layers of GRS, but not in either GRS layer 5 or BF layer 2 (Fig. 8). The localization of Kv4.3 mRNA expression in the superficial layer of GRS has already been shown by others (Serôdio and Rudy 1998). Kv1.1 is known to be widely expressed in neocortex of mouse (Allen Brain Atlas, http://mouse.brain-map.org/), and has been investigated in supragranular pyramidal neurons of rat somatosensory and motor cortices (Guan et al. 2006).

#### Single-cell RT-PCR

**Fig. 9:**
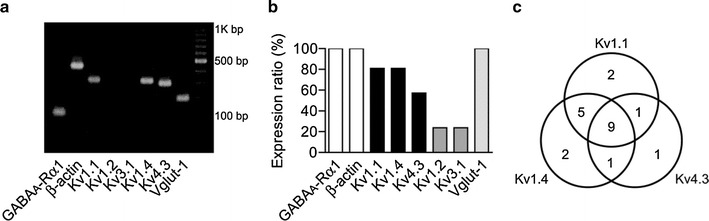
**a** Single-cell RT-PCR for one layer 2 neuron in GRS. Eight genes were tested. Amplified cDNA fragments were confirmed by 2% agarose gel electrophoresis, with a 100 bp DNA ladder marker. Note the presence of genes for Kv1.1, Kv1.4 and Kv4.3. For this cell, genes for Kv1.2 and Kv3.1 were not found (*two dark lanes*). **b** Expression ratio of each of these genes in 21 GABA_A_ receptor α1 subunit and β-actin positive LS neurons. **c** Venn diagram showing the number of cells expressing Kv1.1, Kv1.4 and Kv4.3 messages. Note that all of the LS neurons (*n* = 21) expressed at least one of three Kv channel messages

Microarray and in situ data provide only laminar-specific resolution. We next employed single-cell RT-PCR to assess potassium channel mRNAs across individual LS pyramidal neurons in layer 2 of GRS (Fig. 9a). Expression of β-actin and GABA_A_ receptor α1 subunit was used as a control for the experimental accuracy for a given cell. With this criterion, results were obtained for 21 LS neurons (Fig. 9b). Five Kv channel mRNAs were screened: Kv1.4 and Kv4.3, and for comparison Kv3.1. We also examined Kv1.1 and Kv1.2 expression in addition to Kv1.4 and Kv4.3, because Kv1.1 and Kv1.2 containing potassium channels have been reported to be responsible for LS firing property in medium spiny neurons in rat striatum (Shen et al. 2004) and superior colliculus (Saito and Isa 2000). Of these five channels, Kv1.1 and Kv1.4 mRNAs showed the highest expression ratio (81% each, *n* = 17). Kv1.1 and Kv1.4 were co-expressed in 14 neurons, and were independently expressed in 6 neurons (3 for Kv1.1 and 3 for Kv1.4). In total, 20 out of 21 LS neurons expressed Kv1.1 and/or Kv1.4 mRNAs. Kv4.3 mRNA was detected in the remaining one LS neuron. This cell was included in 12 LS neurons (57% of 21) expressing Kv4.3 mRNA. Of those, nine neurons co-expressed Kv1.1 and Kv1.4 mRNAs (see Venn diagram, Fig. 9c).

The expression ratios for Kv1.2 and Kv3.1 were rather low (24% each; Fig. 9b). These findings are consistent with the data from gene chip and/or in situ hybridization, and point to a preferential role for the Kv 1.1, Kv1.4 and Kv4.3 channels in the generation of the LS property.

We note that VGluT1 mRNA was expressed in all the screened neurons, consistent with other evidence that LS neurons are glutamatergic pyramidal neurons.

#### Pharmacology

**Fig. 10:**
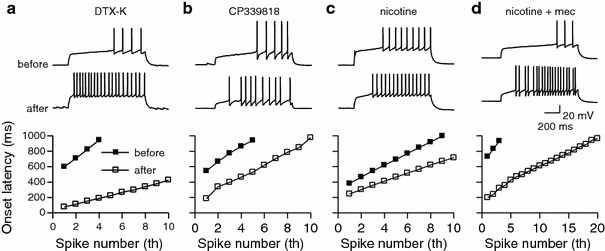
**a** A representative example showing the effect of 100 nM DTX-K on the LS firing pattern. Voltage responses to a depolarizing current for a layer 2 LS neuron, before and 10 min after the administration of DTX-K are shown. The *lower graph* shows the change in the onset latency of spikes in the same LS neuron. Note that the onset latency of the first spike was reduced from 660 to 80 ms after DTX-K application. **b** Similar to **a**, showing the effect of 3 μM CP-339818. **c** Similar to **a**, showing the effect of 100 nM nicotine. **d** Similar to **a** showing the effect of 100 nM nicotine in the presence of mecamylamine (10 μM)

The contribution of KV1.1, Kv1.4 and Kv4.3 channels to the LS property was further tested by pharmacological blockade of these channels (Fig. 10). In the presence of 100 nM dendrotoxin-K, a specific blocker for Kv1.1, the latency of the first spike evoked by just-super threshold current was shortened to 24 ± 2.2% (*n* = 5, Fig. 10a). At this concentration, DTX-K slightly depolarized the resting membrane potential and decreased the input resistance in some cases. In such cases, the resting potential was brought back to the original level by DC current injection and the amplitude of depolarizing current pulse was increased to just-super threshold level. In the presence of 1–3 μM CP-339818, a blocker for Kv1.4 channels at this concentration (Nguyen et al. 1996), the latency of the first spike evoked by just-super threshold current was shortened to 54 ± 7.6% (*n* = 8, Fig. 10b) without a change in the resting membrane potential and the input resistance. The drug did not affect the first spike latency in 3 out of 11 LS neurons tested. Although CP-339818 also blocks Kv1.3, we concluded that the drug specifically blocked Kv1.4 channels in our experimental condition, because the Gene-chip analysis showed that the expression of Kv1.3 was low in GRS layer 2. Nicotine (100 nM), reported to block Kv4.3 channels directly in cardiac muscle (Wang et al. 2000), also shortened the onset delay of the first spike evoked in response to a depolarizing current injection (48 ± 3.5%, *n* = 5, Fig. 10c). In the presence of mecamylamine (10 μM), a nonselective blocker for nicotinic acetylcholine receptors, we obtained a similar result (53 ± 9.9%, *n* = 5, Fig. 10d), confirming that nicotine directly suppressed Kv4.3 channels, as in the case of cardiac muscle (Wang et al. 2000). The firing pattern of 3 out of 8 LS neurons was not affected by nicotine. Since some LS neurons were not affected by CP-339818 or nicotine, additional factors might be contributing to the LS firing property (see “Discussion”).

### Area specificity of LS neurons

We carried out a short survey of two other areas interconnected with GRS for the presence of LS neurons; namely, the presubiculum and perirhinal cortex. In the presubiculum, nine neurons were recorded from the superficial layer. Five of these were classified as RS, three as LS, and one as LS-like. In perirhinal cortex, neurons were identified as mainly RS (12 of 18 neurons from layer 2, and 7 of 12 neurons from layer 6). Of the 6 other layer 2 neurons, 1 was LS, 1 stuttering, 3 FS, and 1 single spiking. In the layer 6 sample, 1 neuron was LS, 1 FS, and 3 intrinsically bursting.

## Discussion

The LS property is unusual for cortical pyramidal neurons, but has been previously described for several other cell types, in particular: medium spiny stellate neurons of the basal ganglia (Nisenbaum et al. 1994), neurons in the intermediate layers of the superior colliculus (Saito and Isa 2000), and cortical neurogliaform cells (Kawaguchi 1995; Chu et al. 2003). In barrel cortex, the delay in firing of neurogliaform cells may be associated with a slow initiation of whisker-evoked action potentials (Zhu et al. 2004).

Pyramidal cells in rat perirhinal cortex have been reported to exhibit long delays in spike initiation when injected with depolarizing current steps. Encoding over long time intervals would be appropriate to associative learning, a function identified with perirhinal cortex (Beggs et al. 2000; McGann et al. 2001). LS neurons in perirhinal cortex were reported in high percentages in both the upper layers (19 of 26 neurons) and layer 6 (74 of 86 neurons). In our screen of perirhinal cortex, we identified two neurons as late spiking (one in layer 2, from a total of 18; one in layer 6 from a total of 12). Other investigators have identified perirhinal neurons in the upper layers as behaving as RS relay cells (Biella et al. 2007). The apparent discrepancy in the abundance of LS neurons is surprising but may be due to sublaminar or other variations in the location of the recording sites, and/or differences in the recording conditions.

What is the significance of LS neurons in the rat GRS? In previous discussions, the LS property has been interpreted as required for synaptic integration in a certain context. The GRS is known to contain head-direction cells (Chen et al. 1994; Cho and Sharp 2001), and has been implicated in aspects of learning and memory in a wide range of behaviors such as visual and vestibular integration, path integration, and spatial navigation (Cooper and Mizumori 1999, 2001; Cooper et al. 2001; Harker and Whishaw 2002; Vann and Aggleton 2002; Vann et al. 2003; van Groen et al. 2004). This functional profile could be consistent with a LS property, where synaptic inputs are compared or integrated during the delay interval. On the other hand, it is clear that the LS property per se is associated with a range of neuronal phenotypes, including both GABAergic and glutamatergic neurons in several different regions (see above). The exact mechanism and significance of the LS property may depend on area-specific circuitry.

Our results on the physiological distinctness of GRS layer 2 pyramids, are in accord with several recent studies that demonstrate layer-specific dysregulation in the GRS after anterior thalamic lesions (Poirier and Aggleton 2009; Amin et al. 2010). That is, activity-related markers show either decreased levels in superficial GRS (*c-fos*, zif268, 5ht2rc, kcnab2) or, for one marker (cox6b), increased levels (Amin et al. 2010). These results highlight the role of GRS as part of an extended network, notably involving the anterior thalamic nucleus and the hippocampal formation. The distal dendritic tufts of layer 2 neurons, in upper layer 1, are potential postsynaptic candidates of projections from the anteroventral thalamus (Shibata 1993), which converge in this layer with GABAergic input from CA1 hippocampus (Miyashita and Rockland 2007).

The GRS could have multiple roles in an extended thalamic-hippocampal network. Working memory tasks, for example, increase immediate early gene activity in the GRS during spatial learning and navigation based on both internal and external cues (light and dark conditions; Pothuizen et al. 2009). No layer-specific patterns were identified in these experiments. However, another recent experiment has reported a selective change in* c-fos* expression in layer 2 GRS neurons during a spatial, but not during a non-spatial version of the Morris water maze in mice (Czajkowski et al. 2008).

Another possibility is that the LS neurons as a population are involved in synchronous activity. The GRS is recurrently interconnected with hippocampal structures, and is known to be one of several regions which can independently generate theta-range oscillations (Kirk and Mackay 2003; Talk et al. 2004). Synchronous firing could be achieved by extensive axonal interconnectivity, such as has been demonstrated for LS neurogliaform neurons in cortical layer 1 (Chu et al. 2003). However, although GRS neurons in layer 2 have horizontal, intralaminar collaterals, these are not unusually abundant, nor are collaterals restricted to this layer. The conspicuous bundling of apical dendrites is another candidate mechanism for synchronous firing, either via direct dendro-dendritic appositions among the apical dendrites or via the intermingled parvalbumin-positive dendrites (Ichinohe and Rockland 2002). In a previous electron microscopic study (Ichinohe et al. 2003), we reported direct appositions in the upper layers between distal dendrites of layer 5 GRS neurons, identified by immunolabeling for OCAM. In that study, appositions between putative layer 2 apical dendritic trunks were observed in single sections, but this observation requires confirmation with serial section reconstruction or identifying markers specific for layer 2 apical dendrites. Classical gap junctions were not observed between pyramidal cell dendrites, although these were found between parvalbumin-positive dendrites (Ichinohe et al. 2003).

### Potassium channels

The large family of potassium channels has been extensively investigated; and the expression, distribution, and biophysical properties have been characterized for multiple subunits (Gabel and Nisenbaum 1998; Shen et al. 2004; Guan et al. 2006; Vacher et al. 2008). Single-cell RT-PCR, immunocytochemistry, and whole-cell recordings with specific peptide toxins have established that neocortical pyramidal cells express multiple delayed rectifier Kv1 α-subunits, likely to play a role in regulating cell excitability (Guan et al. 2006, 2007).

We have demonstrated in this report that Kv1.1, Kv1.4 and Kv4.3 channels are highly expressed in layer 2 pyramidal neurons in GRS and provided evidence for their contribution to the LS firing property in those pyramids. Kv1.1 is classified as a delayed rectifier, while Kv1.4 and Kv4.3 generate the so-called “A-type” rapidly inactivating potassium currents (*I*_A_). Our single-cell RT-PCR experiment showed that the Kv1.1 channels were expressed in the LS neurons in layer 2 of GRS, but not selectively for this layer and cell population (Table 2). Indeed, it has been reported that Kv1.1 channels are widely expressed in supragranular neocortical pyramidal neurons (Guan et al. 2006). The firing property of these neurons is mostly RS (McCormick et al. 1985; Sutor and Hablitz 1989; Mason and Larkman 1990; Cho et al. 2004), as was found in our present results (Fig. 6). However, Kv1.1- and Kv1.2-containing potassium channels have been proposed to regulate the LS firing property in striatal medium spiny neurons (Nisenbaum et al. 1994; Gabel and Nisenbaum 1998; Shen et al. 2004). Correspondingly, we found that application of dendrotoxin-K (100 nM), specific blocker for the Kv1.1 channel at this concentration, reduced the onset latency of the first spike in layer 2 LS neurons of GRS (Fig. 10). Our pharmacological experiments also show effects of *I*_A_ in layer 2 LS pyramids. This current has been attributed to several combinations of Kv channels (Gabel and Nisenbaum 1998). In cultured *Drosophila* neurons, *Sh* and *Shal* channels, mediating *I*_A_-like rapidly inactivating currents, have been reported to contribute to the formation of a “delayed” firing property. Interaction of rapidly and slowly inactivating currents has been implicated in specific firing patterns in *Drosophila* (Peng and Wu 2007). In developing and cultured cerebellar granule cells, suppression of *I*_A_ by expressing a dominant negative mutant Kv4.2 resulted in shortening of latency before the first spike generation (Shibata et al. 2000). Whether the same channel combinations characterize LS layer 3 pyramidal cells requires further investigation.

In summary, by microarray, in situ hybridization, and single-cell RT-PCR, we showed a clear association of delayed rectifier and A-type potassium channels, Kv1.1, Kv1.4 and Kv4.3, with neurons in layer 2 of the GRS. Further experiments will be needed to determine whether these channels additionally contribute to other properties (e.g., Kv4.3-mediated currents as underlying rhythmic activity in hippocampal interneurons, Bourdeau et al. 2007), and to determine the full panoply of channel interactions responsible for the LS firing property.

## Conclusion

In conclusion, layer 2 GRS neurons in the rat are a distinctive population, with a common output (i.e., callosally projecting; Wyss et al. 1990), strong apical dendritic bundling, and shared unusual firing properties. A recent study reports that layer 2 GRS neurons are developmentally distinctive, characterized by late migration from the subventricular zone during the first postnatal week (Zgraggen et al. 2011). Some layer 3 neurons have callosal outputs and show LS firing properties, although layer 3 neurons have a slightly different dendritic morphology. How these features subserve aspects of learning and memory remains for further investigations.
